# Motives for Dropout Among Former Junior Elite Caribbean Track and Field Athletes: A Qualitative Investigation

**DOI:** 10.3389/fspor.2021.696205

**Published:** 2021-07-09

**Authors:** Candice E. Thomas, Timothy P. Chambers, Luana C. Main, Paul B. Gastin

**Affiliations:** ^1^School of Exercise and Nutrition Sciences, Deakin University, Geelong, VIC, Australia; ^2^School of Psychology, Deakin University, Geelong, VIC, Australia; ^3^La Trobe Sport and Exercise Medicine Research Centre, School of Allied Health, Human Services and Sport, La Trobe University, Melbourne, VIC, Australia

**Keywords:** athlete burnout, junior-elite, motivational atmosphere, psychosocial influences, talent development

## Abstract

Anecdotal reports within the Caribbean track and field fraternity have revealed that there is a high level of athlete dropout from competitive sport at the junior-elite level, and a poor transition to senior-elite status. Consequently, this qualitative investigation explored the key motives that may have contributed toward the unsuccessful transitions and ensuing dropout of Caribbean track and field athletes during the junior to senior transition period. Eleven former junior-elite track and field athletes (four males, seven females; M_age_ = 29, SD ± 4.2 years) from four English-speaking Caribbean islands participated in semi-structured interviews. Following an inductive and deductive thematic analysis, four higher order themes were identified: (1) “there's not enough support”; (2) “felt pressure to make sure I committed”; (3) “it's always competitive here”; and (4) “battle with the injuries.” For these former junior-elite Caribbean athletes, the decision on whether to continue within the sport was influenced by a combination of factors, although inadequate financial and organizational support had the most bearing on athletes' decision to drop out during the crucial transition years. Implications for consideration by key stakeholders and policymakers within the region are discussed.

## Introduction

The Caribbean sport ecosystem represents a complex network of local sporting organizations (e.g., government—ministry of sport, sport federations, sporting high schools, and private clubs etc.) and individuals (managers, sport administrators, coaches etc.) interacting with each other within the Caribbean athlete development pathway (Thomas et al., 2019). Significant limitations have been reported within the development pathway stemming from the historical economic prioritization by governments on other key sectors in the region (e.g., mineral extraction, agriculture and tourism) (Mc Cree, 2002). Consequently, investment, policy formulation and planning in sport at the state and private sector levels have not been prioritized (Kaufman et al., 2013). As a result, there exists significant underdevelopment in sporting facilities, financing, sport governance and sport professionalization within the Caribbean region which adversely affects the development of young talented athletes (Mc Cree, 2002; Kaufman et al., 2013). The sport of track and field is one of the premier sports practiced within the Caribbean region with some countries showing greater dominance over others. Notwithstanding some of the aforementioned limitations within the sport development pathway, Caribbean athletes have experienced a great degree of success in the sport of track and field over many decades at the highest levels of competition (i.e., the Olympic Games and World Championships), the most notable being Usain Bolt (Irving, 2010).

Athletic career development and transitions research has evolved over the last three decades, with greater emphasis being placed on athletes' within-career transitions and the adoption of a whole person approach by exploring a range of contextual factors influencing athletes engagement and progression within sport (Stambulova et al., 2009). Among the available career development frameworks, the holistic athletic career model (HAC) (Wylleman et al., 2013) has been recognized as the most comprehensive. The model highlights the transitions within an athlete's career across four stages: initiation (from 6 to 7 years), development (from 12 to 13 years), mastery (from 18 to 19 years), and discontinuation (from 28 to 30 years) (Wylleman and Rosier, 2016). These transition stages coincide with the athletes' psychological (i.e., childhood, adolescence and young adulthood), psychosocial (i.e., family, peers/partner, coach support), academic/vocational (primary, secondary, tertiary level education/ work) and financial (i.e., material and logistic support) development layers. The age of when the junior to senior transition period starts is difficult to identify as it is said to depend on different factors: gender, type of sport and the sociocultural context (Stambulova and Ryba, 2014). Within this study, the authors used the age range of 17–25 as it represented most Caribbean athletes' transition out of high school on the lower end and transition out of college/university on the upper end. Most pre-existing research that has investigated the junior to senior transitions of athletes has been conducted within developed countries which may limit generalizability of findings to lesser developed nations (Hollings and Hume, 2011; Hollings et al., 2014; Franck et al., 2016, 2018; Stambulova, 2017; Franck and Stambulova, 2019).

In spite of the successes attained at the international level, anecdotal reports from the Caribbean track and field fraternity have revealed that there is a high level of athlete dropout from competitive sport at the junior-elite level, and a poor transition to senior-elite status (Trackandfieldnerdz, 2019). Globally, the junior to senior transition phase is well-known for its high dropout rate with some of the most advanced sporting nations having reported similar trends (Bussmann et al., 1994; Hollings and Hume, 2011; Shibli and Barrett, 2011). The transition from junior to senior level sport has been described as being highly stressful and a difficult transition period for athletes as they adjust to new challenges (Stambulova et al., 2009). For example, changes in social relations and the degree of influence and support from individuals within their social support network (e.g., peers, family, coach) tend to occur during this period (Keegan et al., 2014). Additionally, there is increased pressure on the athlete that can be linked to their inability to cope both mentally and physically to the increased athletic demands required for higher training and performance outcomes (Stambulova, 2017). Compared to adults, adolescents have underdeveloped coping and problem-solving skills, further compounding this already challenging transition (Hampel and Petermann, 2006). Junior athletes are also challenged with the demand to find an optimal life-balance between sport and other activities (e.g., academic and social activities) (Stambulova et al., 2015). An unsuccessful transition is said to occur as a result of the ineffective coping of athletes in relation to the increased demands and stressors which has been associated with athlete dropout (Stambulova, 2017). In this study, dropout is defined as premature termination of a sport career before the athlete could reach individual peak performance.

Factors influencing young athletes' motives to drop out of competitive sport have been widely discussed within the research literature (Bennie and O'Connor, 2006; Enoksen, 2011; Balish et al., 2014; Hollings et al., 2014; Crane and Temple, 2015). The most recent systematic review of factors associated with dropout of organized sport among children and adolescents revealed that intrapersonal (e.g., lack of enjoyment, perceptions of competence) and interpersonal (e.g., social pressures, competing priorities) constraints were most frequently associated with discontinuation (Crane and Temple, 2015). These findings correspond with the premise of the self-determination theory which underscores that when the environment is supportive of an athlete's basic needs, they experience a heightened sense of autonomy (Ryan and Deci, 2000) competence (White, 1959) and relatedness (Baumeister and Leary, 1995). It is considered that satisfying these basic psychological needs fosters wellbeing and strengthens inner resources (e.g., resilience) resulting in behavioral and emotional responses conducive to long-term participation in the sport (Ryan and Deci, 2000). Alternatively, if the satisfaction of the basic needs is not met, frustration ensues and evokes ill-being and increases vulnerabilities which can lead to disengagement (Ryan and Deci, 2000). A key limitation noted by authors of this systematic review were that studies had neglected to include the broader societal contexts such as socio-economic status, sport organization policies or the interactions between these broad environmental factors and the individual (Crane and Temple, 2015). Additionally, the authors highlighted a general lack of examination of the complexity and interrelationship between dropout factors within research, and underscored the lack of investigation of children's vs. adolescent's reasons for dropout (Crane and Temple, 2015).

Anecdotal reports from within the Caribbean region describe a high attrition of young talented track and field athletes and criticize the poor transitions of junior-elite athletes to the senior-elite level. However, limited research has explored the key motives that contribute to the unsuccessful transitions and ensuing dropout of junior-elite track and field athletes within developing countries, and none within the Caribbean context. To date, the vast majority of transition and dropout studies have been conducted in North America, Europe and Australia which are characterized as high income nations (Crane and Temple, 2015). This may limit applicability and understanding of dropout within less developed sporting nations such as those of the Caribbean region where significant underdevelopment in the sporting sector has been reported (Mc Cree, 2002; Kaufman et al., 2013). In order to build on the successes attained in athletics within the Caribbean region it is imperative that the reasoning behind junior-elite athlete transitions and dropout is fully explored. Consequently, the purpose of this study was to explore the key motives that may have contributed toward the unsuccessful transitions and ensuing dropout of former junior-elite Caribbean track and field athletes from competitive sport during transition to senior competition. More specifically, the following question sought to solicit information through the athletes' experiences: What perceived motives influenced the decision of former junior-elite track and field Caribbean athletes to discontinue competitive sport during the junior-senior transition years?

## Methodology

### Approach

The study was underpinned by the holistic athletic career model (HAC) (Wylleman et al., 2013). This study was grounded in an interpretivist philosophy, underpinned by ontological relativism and epistemological constructionism. The relativist ontological position allowed the authors to consider participants reality to be multiple, contextual, and denoted by the meanings of the social world based on their experiences within this context and in relation to others (Thorpe and Olive, 2016). The epistemological constructionist approach, meant that the authors acknowledged that the research process, including our interpretations of the participants meaning-making, were shaped by our social identities, our lenses and backgrounds as coaching and sport science practitioners, researchers in sport and exercise science, and our personal experiences engaging in elite sport (Sparkes and Smith, 2013). Given the importance of culture in the Caribbean, the authors believe that the constructionist approach is extremely valuable as it positions us to think that each individual athlete experience is bound within their cultural existence.

### The Research Team

In line with an interpretivist paradigm, it is important to note the roles and experiences of the research team. The first author was responsible for conducting all of the interviews and lead the data analysis and writing. Moreover, the first author was a former Caribbean track athlete and has more than a decade of experience as a professional track and field coach of both junior and senior athletes within the Caribbean. The desire to understand athletes' experiences during the crucial junior to senior level transition period and the longer-term desire to generate stop gap measures to minimize the attrition in Caribbean track and field was derived from her applied experiences. The second author acted as a critical friend (Smith and McGannon, 2018) throughout the analysis and has over 10 years of experience in sport and exercise psychology and the provision of athlete support services. The third and fourth authors were responsible for developing the research idea and questions together with the first author and providing higher-level feedback on the thematic structure where all themes were collectively reviewed. The third and fourth authors have over 40 years of combined experience in applied sport science, athlete monitoring, high performance management and system development.

### Participants

The participants in this study were 11 former junior-elite track and field athletes (four males and seven females) from four English-speaking Caribbean islands (Jamaica, Trinidad, Tobago, Dominica). The mean age of participants at the time of the study was 29 years (SD ± 4.2 years). The mean age of withdrawal from competitive sport for participants was 22 years (SD ± 2.2 years) (see Table 1). All participants had competed or medalled at least once in the Caribbean Junior Championships (CARIFTA), the Central American and Caribbean Games (CAC) and/or the World Junior Championships (WJC) (between 1998 and 2014). The CARIFTA Championships and CAC Junior Games are the two principal regional and international track and field competitions through which the majority of national junior (Under17 and Under 20) representatives from Caribbean and Central American nations participate en-route to senior level competition. More specifically, the CARIFTA Games is considered a rite of passage at the junior level for almost all Caribbean elite track and field athletes (e.g., Usain Bolt−3-time CARIFTA gold medallist). Of the listed participants, 54% attended university post high-school with no sporting scholarship, 36% attended university with a sporting scholarship and 10% did not attend university. Athletes specialized in one of the following track and field disciplines: sprints/hurdles, throws, middle distance and multi-events and were all national junior athletes. In total, participants amassed a total of eight gold medals, six silver medals and 8 bronze medals across the international Championships/Games at the junior level.

**Table 1:** Identifies each athlete using a number (A1–A11), and provides a summary of their demographic information and primary reason for sport withdrawal.

**Athlete**	**Gender**	**Age (at interview)**	**Age at withdrawal**	**Highest educational level attained**	**Track and field discipline**	**Primary reason for withdrawal**
A-1	Male	29	25	Tertiary	Throws/Multi-Events	Toll of injury
A-2	Female	19	17	Secondary	Sprints/Hurdles	Perceived Inadequate finances to rehabilitate injury
A-3	Female	31	24	Tertiary	Middle/Long Distance	Toll of injury
A-4	Female	29	22	Tertiary	Throws	Toll of injury
A-5	Male	27	21	Tertiary	Throws	Perceived inadequate finances
A-6	Female	27	22	Tertiary	Sprints/Hurdles	Perceived inadequate finances
A-7	Female	31	23	Tertiary	Sprints/Hurdles	Perceived inadequate finances
A-8	Male	32	25	Tertiary	Sprints/Hurdles	Conflicting commitments
A-9	Female	35	23	Tertiary	Middle/Long distance	Perceived inadequate family and organizational support
A-10	Female	31	23	Tertiary	Sprints/Hurdles	Conflicting commitments
A-11	Male	25	21	Tertiary	Multi-events	Perceived inadequate organizational support

### Data Collection

Ethics approval was obtained from the first author's higher education institution. The first author's background as a professional track and field coach within the Caribbean facilitated the recruitment of participants who were purposively sampled through professional contacts. The criteria used to select the participants for this study included: (a) having been a junior-elite level track and field athlete and having discontinued competitive sport on/before the age of 25; (b) having competed or medalled at an international junior level competition; (c) being an athlete from an English-speaking Caribbean country. The junior-elite classification was defined as a junior athlete (≤ 20 years) that has competed or medalled at an international elite level track and field competition. All interviews were conducted by the first author. All participants completed a demographic questionnaire (e.g., age, country, school attended etc.) before the commencement of each interview. Interview dates were agreed at a convenient time and an informed consent was signed by each participant on the date of the interview. The interviews were between 36 and 80 min (Mean 56 SD± 14 min). Eleven interviews were conducted, inclusive of five face-to-face interviews, and six video conferencing interviews due to geographical location. All interviews were digitally recorded and were transcribed verbatim.

The semi-structured interview guide was grounded on previous literature on athlete development and transitions in sport (Côté et al., 2007; Wylleman et al., 2013). The interview began with a general discussion of the participant's background information as listed within the demographic questionnaire (e.g., “What primary/secondary school or university did you attend etc.?”), to enable the participant to become comfortable with the interviewee and research process. The ordering of questions was deliberate such that the questions initially focused on participants' sporting activities and psychosocial influences at the development stage and progressed to their experiences surrounding their transition to the early mastery stage. The interviews started by asking the participants to describe their involvement in sport (e.g., “How did you become involved in track and field?”). Next questions moved into a discussion on the key social influences during the development and mastery stages and the types and volume of training they underwent during these stages (e.g., “Can you describe the types and volume of training that you underwent at the secondary/ tertiary level?”). The interviews then moved onto the main questions regarding the benefits and challenges associated with the balancing of sport, school and family (e.g., ‘As a young athlete, what was it like juggling/balancing sport, school and family commitments?'). Penultimate questions focused on the factors that participants thought influenced their decision to discontinue training and competing in high-performance sport? (e.g., ‘What factors influenced your decision to discontinue training and competing in high performance sport?'). Finally, interviews ended with questions summarizing the key ideas identified earlier in the interview. Probes such as “could you explain in more detail?” were used throughout the interview to clarify or solicit more complete answers (Neuman, 2007).

### Data Analysis

As applied practitioners and researchers, the authors endeavored to employ an analytical strategy that enabled them to reflect on their collective experiences as they pertained to the collection and analysis of data. More congruent with the “big Q” qualitative approach, the authors chose to pursue a strand of thematic analysis that was atheoretical (Braun et al., 2016) such that the analysis aligned with our philosophical assumptions. Accordingly, we believe that our analysis is congruent with reflexive thematic analysis (Braun and Clarke, 2019) as we pursued a deductive and latent approach to the construction of themes.

For qualitative researchers, there is a notable risk of appearing to adopt a recipe-like analytical process (Willig, 2013). We wanted to remain genuine and authentic to the reflexive and flexible nature of thematic analysis, hence we navigated the following phases (Braun et al., 2016). First, all data were transcribed by both the first author and an external transcription company. Second, the first author familiarized herself with the data and listened to each interview at least once, and subsequently read and re-read all transcripts. Third, once the first author felt sufficiently immersed in the data, she then proceeded to generate preliminary codes (e.g., words or phrases used by participants, labels relating to the research question) in a systematic fashion by highlighting data extracts in color-codes and making general notes on recurring codes. She did this until she perceived that the codes captured patterns of meaning as well as differing perceptions across the data set in relation to the research question. A list of coded data was generated for each participant in which the codes, their perceived meaning and corresponding data extracts were outlined. The second author cross-checked all codes and extracts and provided independent interpretations of the data. Third, the first and second authors compared their interpretations of data, discussed inconsistencies and came to a consensus where interpretations differed. Subsequently, the first author proceeded to cluster preliminary codes into raw data themes, based on feature similarity between codes, before categorizing them into lower order subthemes. Fourth, a thematic map was generated to clarify the sorting process and to represent the relationships between different codes and themes. Each theme was reviewed and refined by revisiting the codes in the context of the original transcripts so that they were congruent with the collated extracts as well as the entire data set. Fifth, higher order themes were subsequently clustered into general dimensions to present a more meaningful and coherent picture of the participants' views. At this point, the authors deliberately attempted to deductively “sort” the themes into four final dimensions that aligned with pre-existing literature on athlete development and transitions e.g., (Bennie and O'Connor, 2006; Côté et al., 2007; Wylleman et al., 2013). The second author assisted throughout this process as a critical friend (Smith and McGannon, 2018) to ensure that various potential interpretations were explored. The third and fourth authors provided higher-level feedback on the thematic structure whereby themes were revisited, reviewed, and revised to better understand their fit and enable refinement (Srivastava and Hopwood, 2009). During the analysis and writing process, the first author moved back and forth flexibly and iteratively between identified themes, codes, and data excerpts, which often stimulated further exploration of the data and re-working of themes and subthemes.

### Trustworthiness

The term “trustworthiness” is used by qualitative researchers to represent quality credibility of the data and interpretation (Sparkes and Smith, 2009; Smith et al., 2014). First, a balanced group of male and female athletes with specific and extensive experience at international junior competitions were “purposively sampled” to achieve substantive contribution and width in relation to the topic area. For example, participants were selected across four track and field disciplines and either belonged to larger territories which possess a more dominant track and field culture and greater resources (e.g., Jamaica, Trinidad) or smaller territories with miniscule populations and lesser resources (e.g., Tobago, Dominica). To improve ‘credibility and reliability' of athlete responses, interview questions touched on actual events and memories (e.g., top junior athletic achievements in competition) in the athlete's development, previously shown to mitigate against recall bias (Côté et al., 2005). Secondly, to enhance ‘methodological rigor' of this research (Smith et al., 2014), prior to commencing data collection, the interview guide was pilot tested with three former junior-elite athletes of similar caliber and age range belonging to the sports of cycling, sailing and taekwondo. This process resulted in the refinement of the interview guide (e.g., reduction in the quantity of questions where there was overlap). Thirdly, elements of the research process were documented in an audit trail of events (e.g., reflexive note taking about meaning and potential patterns in data by the first author). This process aided “aesthetic merit” and “coherence” through the reconfiguring of original codes and themes during the project (Smith et al., 2014). Finally, in this study, quality of analysis and trustworthiness was enhanced by engaging “a critical friend.” The second author served as a “critical friend” in the investigation by encouraging critical dialogue regarding the analysis and interpretation of findings between researchers. Specifically, the second author independently cross-checked all codes and provided independent interpretations of the data, promoted the sharing of findings and challenged the first author's assumptions regarding analysis. Rather than creating consensus between authors, this approach provided a theoretical sounding board for the first author's analysis and encouraged reflection upon, and exploration of, multiple and alternative explanations to the emerging data (Smith and McGannon, 2018).

## Results

Findings in this study represent the collated responses of participants highlighting the key motives perceived to have contributed to their unsuccessful transitions and ensuing withdrawal from competitive track and field. Participants highlighted a combination of challenges experienced during the junior to senior transition period. At the individual level, all participants articulated a defining experience that prompted them to withdraw from sport (see Table 1). In spite of these individual differences, there was much commonality regarding a perceived lack of support (primarily financial and organizational). As such, four higher order themes were constructed that resembled the key factors contributing to participants' eventual dropout during the transition years: (1) “there's not enough support”; (2) “felt pressure to make sure I committed”; (3) “it's always competitive here”; and (4) “battle with the injuries.” The findings presented below are first illustrated through the use of direct quotations of the participants' experiences and culminates with a short vignette of one participant in an effort to show how the identified themes work together to create a withdrawal situation. The name and country of the participant (vignette) has been changed to protect their identity. The quotations from the interviews are shown by abbreviations A-1 to A-11 which represent the generic codes provided to each athlete within this study at the time of data coding (see Table 1).

### “There's Not Enough Support”

This higher order theme was developed to represent the perceived lack of support received by participants from their immediate (e.g., family, peers, and coaches) and wider support system (e.g., sporting organizations/administrations). This higher order theme was comprised of three lower order themes: (1) financial burden; (2) underdeveloped sport ecosystem; (3) lack of social support. Most participants expressed that there was inadequate financial and organizational support received from agents within the support system during the transition period from both the immediate and wider support systems. The collective perception of inadequate support contributed to participants' decision to eventually withdraw from competitive sport.

#### Financial Burden

The majority of participants recounted receiving insufficient funding to continue in the sport following their post high school years. For these athletes, the prospect of having to place a financial burden on their parents or themselves was the primary reason most chose to leave the sport early: “I didn't want to be a burden; my father is the one who works and is the one to finance me in mostly everything. So, I didn't want to put him through another set of therapy and doctor bills” (A-2). The weight of these financial demands of track and field at the elite level coupled with the lack of availability of finances were perceived as demotivating factors: “that additional stress of wondering where my rent money is going to come from, where my meals are going to come from. I could not depend on my parents anymore to support me financially” (A-6). Most participants expressed not being able to access government funding or athletic scholarships to continue within the sport during this period. Student-athletes within the Caribbean region generally complete their high school education at 16–17 years, and usually require an athletic scholarship, professional contract or government funding in the years following high school to remain in the sport. A-6 explained her decision-making process during that transition period:

“Should I invest more time, more money into this? Maybe I'll probably make it to the [senior] elite level where I can get funding from the government? Or maybe I may not. I'll have to struggle for those couple of years…burdening my parents additionally…. although they assisted me with track and with school. Or should I just go on to a career path? I chose that path (A-6)”

Most participants perceived that they would not be able to access financial support from external sources within the sport industry (e.g., government and federation funding, professional sponsorship) unless they attained exceptionally high performance standards at the junior level. The perception discouraged many athletes from pursuing the sport long-term: “I didn't think that I would get the sponsorship from Nike, Puma, Adidas, and other local companies to sponsor me” (A-5). Additionally, these athletes felt that there were no financial incentives for them to go to the next level (senior). For most participants, the lack of financial support from the sport industry, particularly at the critical transition period from sub-elite to elite level, demotivated them to continue in the sport:

“I think there's not enough support, especially for those athletes that are trying to develop. Yes, there'll be support for those that are elite, that break world records and make world teams, but what about those athletes who are now trying to follow in those footsteps? Who are now trying to build themselves up to be that elite athlete? and I think we either neglect or we are very selective in who we choose to support (A-6).”

#### Underdeveloped Sporting Ecosystem

Some participants also highlighted the inadequacies and the lack of efficiency in the functioning of the sport ecosystem within their home countries as another factor that contributed to their growing apathy and subsequent withdrawal from the sport. Within the English-speaking Caribbean, organizations (e.g., government—sport federations) and personnel (e.g., sport managers/administrators) within the sport ecosystem are responsible for the management, administration and programming of sport as well as the construction and operational maintenance of facilities within the region. The inadequacies within these sport organizations were specifically noted with regard to participants' criticisms about the lack of specialized sporting facilities (e.g., the lack of a synthetic track) within some Caribbean countries; a necessity for athletes who seek to train and compete at a world-class level. In cases where specialized facilities were available, some participants decried the lack of access granted to these facilities during the competitive training season due to the relevant organizations' decision to prioritize non-athletic events (e.g., concerts, Carnival events etc.):

“A lot of times the athletes don't have a place to train, the track is closed, they have to go to the savannah or go on grass tracks and train outside on the outside field, especially around Carnival time.” (A-6)

Additionally, the absence of a clear athlete development pathway and lack of a transparency in the funding allocation were noteworthy grievances for participants. Furthermore, participants highlighted the administrative conflicts and lack of professionalism encountered either at the local level with coaches or the national level with team managers. For example, the lack of objective criteria used by national team selectors to select athletes for national team representation in international competitions was also a notable grievance for participants. A-10 expressed that the constant uncertainty felt in the national team selection process contributed to her decision to withdraw from the sport: “you train, and you aim for certain things. You're training for your whole year to qualify for an event; know you have qualified and then have all this politics [preferential treatment given to athletes who have not qualified] go on, so it's almost like you're taking a gamble.” In summary, participants felt demotivated as a consequence of having no place to develop as a world-class athlete.

#### Lack of Social Support

Some participants highlighted that a lack of support from their immediate social support network may have adversely affected their motivation levels during late adolescence and their desire to continue within the sport. In the present investigation, social support entailed receiving both emotional (i.e., others being present to provide comfort and security) and esteem support (i.e., others bolstering an individual's competence or self-esteem) from the participant's immediate circle of influence (e.g., family, coach) that contributed to the participant's athletic development. Some participants expressed having experienced undue stress from parents to excel in their sport and win at competitions, which often left participants frustrated and with little desire to continue within the sport. For these participants there was also parental pressure to balance sport and academics; parents frequently reminded athletes that: “you can't be only about track, you have to be about the books.” (A-7). The management of sport and academics at this stage was challenging as “track was demanding” (A-8) coupled with the added parental pressure to “focus on academics” (A-8). For other athletes, the perceived lack of support from their family manifested as a lack of interest shown in their athletic endeavors (e.g., lack of attendance to competition meets, throwing away of medals): “I remember my mother cleaning out and throwing away my medals. It was a bit discouraging, and heart-breaking.” (A-9). These participants craved the social support of their immediate families and became discouraged and subsequently disengaged from the sport when that support was not felt. A-9 reflected on her experience and sought to explain the reasoning behind her mother's absence during her athletic development:

“My mom, she wasn't very supportive. She never came to any of my meets. As I am an adult now I could understand her frustrations in probably trying to make ends meet [generate finances]…that she really didn't have time.” (A-9)

Within participants' support network, youth coaches emerged as a key influence on participants' at the development and early mastery stages. Some participants voiced the negative experiences in their relations with their coaches and the adverse impact it had on their sport participation decisions. For example, some coaches were described as unsupportive and ignorant toward the participant's opinion and were said to display an authoritarian-like coaching style which did not allow for opinions to be heard.

“It was weird because I felt [Coach] was ignorant. Yes, he probably saw something in me, but I felt like he never used to listen. He would feel like “this is what I tell you to do…you have to do this.”' (A-7).

Participants described their dismay at the poor coach qualities displayed (e.g., coach ignorance, poor communication) and the ‘love-hate relationship” (A-10) they had with their coach which often resulted in continuous conflicts at this stage. As a result of the relational conflicts with coaches, these athletes experienced decreased enjoyment in the sport and a reduced desire to pursue competitive sport long-term. Collectively, the perception that there wasn't enough support for athletes appeared to conspire against their motivation to continue in the sport. Without this support, these athletes chose to cease the pursuit of a track and field career and withdraw from the sport.

### “Felt Pressure to Make Sure I Committed”

This higher order theme referred to the pressure participants experienced when attempting to manage multiple commitments, and the conflict that ensued (e.g., personal, academic and professional interests). Two lower order themes emerged: (1) occupational demands and (2) personal demands.

#### Occupational Demands

Participants described the negative impact that their occupational demands (e.g., academic and, in some cases, occupational work) had on their athletic development. In particular, participants recounted challenging experiences associated with trying to maintain the balance between school/work and sport. In some cases, participants had to make significant sacrifices in their sporting endeavors in order to minimize any potential negative impact on their academic standing.

“I qualified for the commonwealth youth games. I didn't go because that would require 2 weeks out of school, and I didn't want to take that 2 weeks out of school. CXC [Caribbean Examination Council] was the next year, and I wanted to have a strong foundation in my class so that I can get a scholarship to go to university. I was afraid to make that sacrifice because I know there's nothing in sports.” (A-11)

Some participants felt pressured within the school environment to excel: “At a young age I was winning junior champs and ranked top-ten in the world, so I felt pressured to make sure I committed to the track.” (A-7). While others found it difficult to manage both academics and sport at this stage. A-6 described her experience within the school environment:

“It was difficult, especially going into form 4, form 5 [final two levels in high school education], where I had to do CXC [Caribbean Examination Council]. We had a lot…from school, to lessons, to practice, to home, to study. It was very, very stressful.” (A-6).

#### Personal Demands

At this level, the pressure to choose a direction was exacerbated by the perceived pressure to decide on whether participants would pursue academics/work, sport, or should prioritize family and personal life en-route to a better life: “After I graduated from college, it was either I stick with doing track and struggle [be financially unstable] or I use my degree and move on and get a job” (A-6). Some participants felt that the high demands of sport adversely affected their academic performance and intruded on their other life commitments. For example, a high degree of uncertainty regarding their future in track and field was perceived as a major deterrent by participants. Consequently, they decided to focus on the area they felt would be most beneficial to their long-term goals. A-5 explained his decision to withdraw from elite sport:

“Going to world championships and so on…I could see that it took a lot of hard work, yes, and it took time. Now time wasn't a luxury that I had because I probably had to find some way to sustain myself and my family while you know… chasing this dream of being a professional athlete.” (A-5).

### “It's Always Competitive Here”

This higher order theme encompassed the training and competition experiences of participants at the development and early mastery levels. One lower order theme emerged: “1) intensive training and competition mode.

#### Intensive Training and Competition Mode

Participants recounted training and competition experiences during their transition into the post high school years, which in some cases included tertiary level enrolment or a job. The training and competition expectations and standards increased once the participants transitioned to a higher age bracket, where they felt more pressure to win. Moreover, participants perceived a broader culture of competition across the Caribbean region that magnified the pressure to win: “It's always competitive here in Jamaica, the competition is intense…we don't like to lose” (A-5). The difficulty in adjusting to a new level of training and competition demands resulted in some participants buckling under the pressure.

“As I got older, and the competition got a little harder, I kinda didn't like it. I would train…and train hard, but when it was time to compete, I would be like “oh Gosh!”. I think that it was kind of a mental block that I had when I was competing, I would probably psych myself out.” (A-3).

This stage was often characterized by some participants as a period of an excessive increase in training volume and intensity: “Yeah the intensity and quantity [training load] was ridiculous. I used to get plenty injuries.” (A-1). Whilst some viewed this excessive increase in load as necessary, others experienced severe negative consequences (e.g., injury). These consequences were particularly salient for athletes who still competed within multiple sports for their schools and/or clubs. Nonetheless, most eventually dropped the other sports due to the high workload and increased training and competition demands. These participants, who were motivated to compete in several sports, conveyed an inability to cope at times with the increased training demands at this level, which resulted in a host of issues including high absenteeism (from training sessions) because of conflicting schedules, burnout, and recurrent injuries:

“He [Coach] always used to tell me you're good at all [sports] but you need to choose one. But I never paid it any mind. and then that is when the injuries started to come into play. I started to tear my hamstring. I started to get injured from football.” (A-8)

The culmination of these issues and other stressors inevitably led to their definitive withdrawal from track and field.

### “Battle With the Injuries”

This final higher order theme represented the compounding effect that a decline and/or stagnation in performance and recurrent injuries had on the athletes' motivation to persist in making the transition from the junior to senior level in track and field. One lower order theme emerged: (1) demotivation and decline in performances. Most participants conveyed that the toll of injury, coupled with their loss of motivation for the sport, inevitably led to their withdrawal from competitive sport.

#### Demotivation and Decline in Performances

Participants explained a casual-like relationship between injury and withdrawal from sport in that getting injured led to a stagnation or decline in their performances that was difficult to overcome and eventually led to withdrawal from the sport. In some cases, the aforementioned funding limitations ensured that many participants did not receive adequate medical treatment: “I had gotten an ACL [anterior-cruciate ligament] tear that I had on my knee and I didn't have any money or anything to do surgery. I tried afterwards to come back [to competitive sport] but it was hard.” (A-4). Many athletes experienced recurrent bouts of frustration following sub-standard performances post injury: “it was always a battle with the injuries, the injury had a lot to play on why I was thinking that I may not continue.” (A-3). This continual discouragement eventually led to reduced motivation to continue in the sport for many participants. A-1 reflected on his mental state during the period he was injured:

“Sometimes you know when you win you feel happy. But if you're constantly training year-in, year-round and you getting injured, it plays on your mental [mental well-being]. It just wasn't fun anymore, know what I mean?” (A-1)

Consequently, some participants expressed that they lost the love for the sport and that it was no longer fun for them. In most cases, this was linked to the toll of injury and their inability to compete and perform at their full potential. In the end, the lack of progression in the performances of participants and their subsequent decrease in motivation levels and desire to continue influenced their decision to disengage from competitive track and field.

### Vignette

As stated previously, a vignette was created to illustrate how the aforementioned factors combined to influence athletes' decisions to withdraw from competitive track and field. The following vignette is based on a compilation of participant experiences and serves to highlight the interaction between the perceived lack of support, conflicting commitments, the intensive sporting environment, and injury.

Marlon is a 21 years old athlete from Jamaica who recently transitioned to the senior level of athletic competition. Marlon has had good athletic success at the junior level, having medalled annually within the U-16, U-18, and U-20 categories in the 100 m and/or 200 m events at the National Junior Championships and/or CARIFTA Games. Marlon graduated from high-school 2 years ago but suffered a hamstring injury in his final year of competition as a junior. Post high school, he joined a local university club to continue to train and compete as well as pursue tertiary education. Marlon still lives at home with his mother (single parent) as he does not deem himself financially stable enough to move out on his own. Marlon sustained a hamstring injury he suffered a year ago and has experienced a decline in performance, meaning that he no longer classifies for elite funding from the Government. As such, lately, he has not been able to meet the requisite performance standard to classify for elite funding. Consequently, he has commenced a part-time job to off-set some of the tuition and medical costs associated with the injury. Although he is receiving some support from the university club through a partial scholarship, it is insufficient to cover his physiotherapy costs associated with his rehabilitation. This has resulted in a significant accumulation of bills. He does not want to ask his mother for assistance as she has supported him financially throughout his junior career, and therefore he feels greatly indebted toward her. Marlon has grown increasingly stressed about his lackluster performances due to the injury and his inability to be competitive in his field. Furthermore, he has become frustrated with the lack of support being provided to developing elite athletes by the government and local sport federation and has begun to contemplate whether it is worthwhile to continue to pursue his Olympic dream of 1 day representing his country like Usain Bolt. Marlon's stagnated performances and accumulating rehabilitation costs coupled with the challenges of balancing personal, work and academic commitments has eventually resulted in him making the decision to officially withdraw from competitive track and field.

## Discussion

This study sought to explore the key motives that may have contributed toward the unsuccessful athletic transitions and subsequent dropout of former junior-elite Caribbean track and field athletes from high performance sport during the crucial transition years. Following interviews with 11 athletes, the authors constructed four higher order themes from the collected data: (1) “there's not enough support”; (2) “felt pressure to make sure I committed”; (3) “it's always competitive here”; and (4) “battle with the injuries.” These findings make a unique contribution to the existing literature which will be discussed below.

### Inadequate Support System

A key finding in the current study revealed that most participants primarily attributed their withdrawal from competitive sport due to the perceived lack of financial and organizational support during the junior to senior level transition years. Previous research has underscored the immense pressure felt by athletes to sustain financial security post-secondary school as a result of expectations that as adults they should now be responsible for their own lives (Riemer et al., 2000; Bennie and O'Connor, 2006). In most cases the former athletes' economic security began to outweigh the importance of continuing to perform at the elite level (Bennie and O'Connor, 2006), thereby forcing the decision between a sporting career, furthering their education, or pursuing a career in the workforce (Hollings et al., 1997). Furthermore, in more advanced sporting nations, talent development is more systematic and individualized and is conducted through specialized organizations that provide holistic support to promising athletes (e.g., Australian Institute of Sport, ASPIRE in Qatar and the English Institute of Sport) (Vaeyens et al., 2008). However, within the Commonwealth Caribbean, there are no talent development academies/institutes (Thomas et al., 2020a,b). Therefore, athletes classified as junior-elite are not placed within specialized talent programs but remain part of the larger group of developing athletes, thus limiting individualized and state sponsored support (Thomas et al., 2019). This issue is further exacerbated by the historical sport development limitations reported within the Caribbean sport sector (Mc Cree, 2002; Kaufman et al., 2013). Although perceived inadequate financial and organizational support during the transition period was the primary reasons reported by most athletes in this study, it was not consistent with previous research that has examined the principal dropout reasons of children and adolescents from organized sport. In comparison, Crane and Temple (2015) systematic review revealed that intrapersonal (e.g., lack of enjoyment and perceived competence), intrapersonal (e.g., social pressures, competing priorities) and structural (injuries) factors were reported as the key factors associated with the dropout decisions of young athletes.

Another finding revealed that some participants perceived that their immediate social support network (i.e., parents, coaches) had an adverse effect on their sport participation decisions during the transition years. These findings were in line with some previous research on the potential negative impact of unsupportive and controlling parental behaviors on young athletes' development and progression in sport (Udry et al., 1997; Holt et al., 2009; Dubuc et al., 2010). More recent research examining the dual-career experiences of late-adolescent athletes has also emphasized the importance of the external family support for junior athletes at this stage in the management of the demands encountered (Baron-Thiene and Alfermann, 2015; Stambulova et al., 2015). Similarly, research on coach-athlete relationships revealed that athlete's motivational levels were significantly influenced by how athletes perceived the instruction, affective responses and leadership style of their coaches (Keegan et al., 2010, 2014; Jowett and Shanmugam, 2016; Jowett, 2017). In fact, controlling coach behaviors have been shown to undermine athletes' self-determined motivation while autonomy-supportive behaviors have been shown to promote it (Gagne, 2003; Amorose, 2007; Gillet et al., 2010). The negative impact of poor coaching behavior on young athletes' sport participation decisions has also been reported in dropout junior-elite track and field athletes from Australia which found that lack of support from coaching staff served as an additional force that influenced their decision to withdraw from the sport (Bennie and O'Connor, 2006). The present results expand upon previous findings by providing further evidence of the impact of an athlete's immediate social support on their sport participation decisions; more specifically within less developed sporting countries.

### Negative Influence of Conflicting Commitments

Findings from the current study also showed that perceived commitment conflicts between sport and academic and work demands during the transition period contributed to the athletes' decisions to withdraw. The findings were consistent with previous research on the dual-career experiences of competitive athletes, which have emphasized the myriad of challenges that co-exist with the increased athletic and academic/work demands during this stage (McCormack and Walseth, 2013; Knight et al., 2018). To compensate for this tension, an effective support network (i.e., parents, teachers, coaches, and organizational support staff) is recommended as it has been shown to be particularly important for continued success and engagement within high performance sport (Knight et al., 2018). However, for those participants in the present study who reported an absence of such a support network, the conflicting commitments appeared to exacerbate the challenges they faced to transition to the next level. Specifically, the lack of access and availability of tertiary level institutions that accommodated student-athletes within the Caribbean context appeared to contribute to their decision to withdraw from the sport.

Although there are some noted challenges associated with dual-career engagement, athletes experience many individual and societal benefits when pursuing a dual-career. Olympic athletes who simultaneously gained academic qualifications and pursued dual-careers were found to be better prepared to manage transitions, cope with expected and unexpected exits from sport, and had the potential to make a positive contribution to the workforce beyond sport (Torregrosa et al., 2015). In addition, positive performance consequences have also been reported within the research literature when there is simultaneous engagement in sport and academia in elite student-athletes (Aquilina, 2013). That the participants in the current study struggled to pursue a dual career suggests that this kind of endeavor is a challenging undertaking within the Caribbean context, with the aforementioned support issues appearing to undermine this kind of pursuit.

### Negative Influence of Intensive Sporting Environment

Findings in this study further highlighted the perceived inability of some participants to cope with the increased training and competition demands during the transition period which led to increased stress and reduced motivation to continue within the sport. Previous research have reported that junior-elite athletes who have dropped out of competitive sport experienced decreased motivational levels, burnout and injury (Bennie and O'Connor, 2006; DiFiori et al., 2014). The high stress state that some athletes experience has been linked to the high training demands associated with elite sport such as traveling, physical requirements and injuries along with the need to balance academics and other life stressors (Gould and Udry, 1996). However, managing such demands may be especially challenging for adolescents, since their coping and problem-solving skills are undeveloped when compared with adults, thus increasing the risk of maladaptive behavioral responses (Hampel and Petermann, 2006). The potential adverse impact of external stressors on athletes is further supported in the research literature in relation to the stress-injury model proposed by Andersen and Williams (1988). The theoretical model hypothesizes that individuals with a history of many stressors and personality characteristics that tend to exacerbate the stress response in addition to having few coping resources (i.e., social support, general coping behaviors) will, when placed in a stressful situation display adverse stress-responses which can lead to higher occurrence of injury (Williams and Andersen, 1998). A positive relationship between high life stress and elevated sport injury risk has been corroborated within the research literature in a wide range of sports and contexts (Williams and Roepke, 1993; Williams and Andersen, 1998; Laux et al., 2015).

The experiences of athletes in this study during the junior to senior transition period were not all negative. In fact, some athletes reported good social support (e.g., supportive coach-athlete and parent-athlete behaviors) as well as positive training and competition experiences during the junior to senior transition years. However, in spite of the aforementioned facilitators, all interviewed athletes were either unable to overcome and/or unwilling to endure the trials and adversities faced during the transition to the senior-elite level. More specifically, it would seem that the perceived inadequate financial and organizational support as experienced by most athletes in this study, served as a huge barrier during the crucial transition period. This, coupled with athletes' inability to cope both physically and mentally with the increased physical and psychological demands encountered (i.e., training and competition, educational and work obligations, personal life commitments, social pressure) proved to be overwhelming. The combination of challenges faced by these athletes may have led to increased stress, reduced motivation and in many cases, the emergence of maladaptive outcomes (e.g., recurrent injuries, burnout) which influenced their decision to discontinue high performance sport in track and field. It should be noted however, that athletes' personal characteristics (e.g., mental toughness, resilience) may have also influenced their sport participation decisions (Vitali et al., 2015; Gerber et al., 2018). Nonetheless, the influence of individual/intrinsic factors on athletes' engagement and progression within the sport was not examined in the current study and is therefore beyond the scope of this paper.

### Implications for Athlete Support

Two important implications for consideration are offered based on the findings in this study. Firstly, the introduction of state sponsored career assistance programs (e.g., financial and sports science/medicine services) is necessary to help sub-elite athletes manage the financial, psychological, social and physical challenges that arise during the junior to senior period. Such programs should ideally be run through a national sport organization/institute with specific responsibilities for elite sport to allow for effective delivery of services to athletes (e.g., English Institute of Sport, Australian Institute of Sport) (Chambers et al., 2013, 2019; Park et al., 2013). Programs should include national “merit” based and tiered support services and funding to allow greater access to state funded sport science and sport medicine services or financial assistance. Additionally, athlete development workshops should be provided with a focus on athletic scholarship opportunities and sub-elite athlete funding requirements. Athlete programs can mimic those currently offered at world leading sport institutes which seek to specifically address the performance needs of developing elite athletes (e.g., English Institute of Sport Performance Lifestyle: https://www.eis2win.co.uk/service/performance-lifestyle/). Secondly, it is important that national governing bodies/sporting institutes provide continuous educational/developmental programs for coaches and parents to improve their knowledge, attitudes and skills in providing autonomy-supportive environments for athletes (Thrower et al., 2017; Turnnidge and Côté, 2017). This would allow for the development of coping skills in young athletes' to mitigate against the increased demands associated with the junior to senior transition period. If developing athletes are adequately prepared and supported, many more talented young Caribbean athletes could transition successfully to the senior-elite level.

### Limitations

The current study provides important insights into specific factors perceived to have been influential in the unsuccessful transitions and subsequent dropout decisions of former junior-elite Caribbean track and field athletes, however they should be reviewed in consideration of the following limitations. First, triangulation between parents, coaches and personnel from within the sporting sector would further strengthen the results of this study by providing a more holistic perspective on the challenges encountered by junior-elite track and field athletes in the crucial transition years. Second, the use of retrospective data collection in some research has been prone to recall in-accuracies (Hodges et al., 2007), however other studies have found that athletes can recall habitual and routine activities such as training and competition, as well as pivotal experiences (Côté et al., 2005; Moesch et al., 2011). Therefore, we can assume with some confidence that the athletes involved in this study accurately recounted the factors that may have contributed toward their dropout from high performance sport during the transition years.

## Conclusion

The findings in this study revealed a combination of financial/organizational, athletic/psychological, academic/vocational and psychosocial factors that may have contributed to the unsuccessful transitions and subsequent dropout of former junior-elite Caribbean track and field athletes' from high performance sport. More specifically, the results revealed that the perceived lack of financial and organizational support from within the athletes' support system during the crucial transition years had the greatest bearing on the dropout decisions of junior-elite athletes. This finding is inconsistent with previous research in more developed countries. Furthermore, the current study shows the interrelationships between the aforementioned factors within the Caribbean sport development pathway and the ensuing adverse impact on the sport participation decisions of talented young athletes during their transition to senior competition.

## Data Availability Statement

The original contributions presented in the study are included in the article/supplementary material, further inquiries can be directed to the corresponding author/s.

## Ethics Statement

The studies involving human participants were reviewed and approved by DEAKIN UNIVERSITY HUMAN ETHICS ADVISORY GROUP—HEAG-H 206_2016. Written informed consent to participate in this study was provided by the participants' legal guardian/next of kin.

## Author Contributions

CT: conception of the rationale, study design, data collection, processing, analysis, interpretation, drafting, revision, and submission of manuscript. LM and PG: assisted with the conception of the rationale, study design, assisted with data analysis and interpretation, and read drafts of the manuscript and provided multiple rounds of feedback to the corresponding author with suggested revisions. TC: assisted with study design, data analysis and interpretation, and read drafts of manuscript and provided multiple rounds of feedback to the corresponding author with suggested revisions. All authors contributed to the article and approved the submitted version.

## Conflict of Interest

The authors declare that the research was conducted in the absence of any commercial or financial relationships that could be construed as a potential conflict of interest.
